# A rare case of primary cardiac B cell lymphoma

**DOI:** 10.1186/1749-8090-9-14

**Published:** 2014-01-14

**Authors:** Andreas Habertheuer, Marek Ehrlich, Dominik Wiedemann, Bruno Mora, Claus Rath, Alfred Kocher

**Affiliations:** 1 Department of Cardiac Surgery, Vienna General Hospital, Medical University of Vienna, Waehringer Guertel 18-20, Vienna A-1090, Austria; 2 Department of Cardiothoracic and Vascular Anesthesia and Intensive Care Medicine, Medical University of Vienna, Waehringer Guertel 18-20, Vienna A-1090, Austria

**Keywords:** Primary cardiac neoplasm, Diffuse large B cell lymphoma, Non-Hodgkin’s lymphoma, Superior vena cava syndrome

## Abstract

Primary cardiac lymphomas represent an extremely rare entity of extranodal lymphomas and should be distinguished from secondary cardiac involvement of disseminated lymphomas belonging to the non-Hodgkin’s classification of blood cancers. Only 90 cases have been reported in literature. Presentation of cardiac lymphomas on imaging studies may not be unambiguous since they potentially mimic other cardiac neoplasms including myxomas, angiosarcoma or rhadomyomas and therefore require multimodality cardiac imaging, endomyocardial biopsy, excisional intraoperative biopsy and pericardial fluid cytological evaluation to establish final diagnosis.

Herein we report the case of a 70 y/o immunocompetent Caucasian female with a rapidly progressing superior vena cava syndrome secondary to a large primary cardiac diffuse large B cell lymphoma (NHL lymphoma) almost completely obstructing the right atrium, right ventricle and affecting both mitral and tricuspid valve. The patient had no clinical evidence of disseminated disease and was successfully treated with extensive debulking during open-heart surgery on cardiopulmonary bypass and 6 cycles of rituximab, cyclophosphamide, doxorubicin, vincristine and prednisone chemotherapy (R-CHOP).

## Background

Primary malignant cardiac neoplasms are extremely rare and among those primary lymphomas constitute only a minor entity. By definition primary cardiac lymphomas belong to the non-Hodgkin’s (NHLs) classification of blood cancers since Hodgkin’s lymphomas seldom show extranodal involvement. Based upon the data of 22 large autopsy series reported by McAllister et al., the frequency of primary cardiac tumors is approximately 0.02%, corresponding to 200 tumors in 1 million autopsies [1]. Metastasis to the heart from other primary cancers is 30 times more common [2]. Only 25% of primary cardiac tumors are malignant and of these as few as 1.3% are primary cardiac lymphomas [3]. Only 0.5% of all extranodal lymphomas present as cardiac neoplasms [3]. Malignant primary cardiac tumors have a dismal prognosis with a survival rate of only 10% at 9 to 12 months [4]. In cases of cardiac inflow obstruction at the time of presentation early surgical debulking plays an important role for initial cardiac and hemodynamic stabilization. However, R-CHOP chemotherapy remains the mainstay of medical treatment. While surgical intervention may initially be necessary to maintain cardiac function the treatment modality most likely to impact the natural history of the disease is R-CHOP chemotherapy [4].

## Case presentation

**Figure 1:**
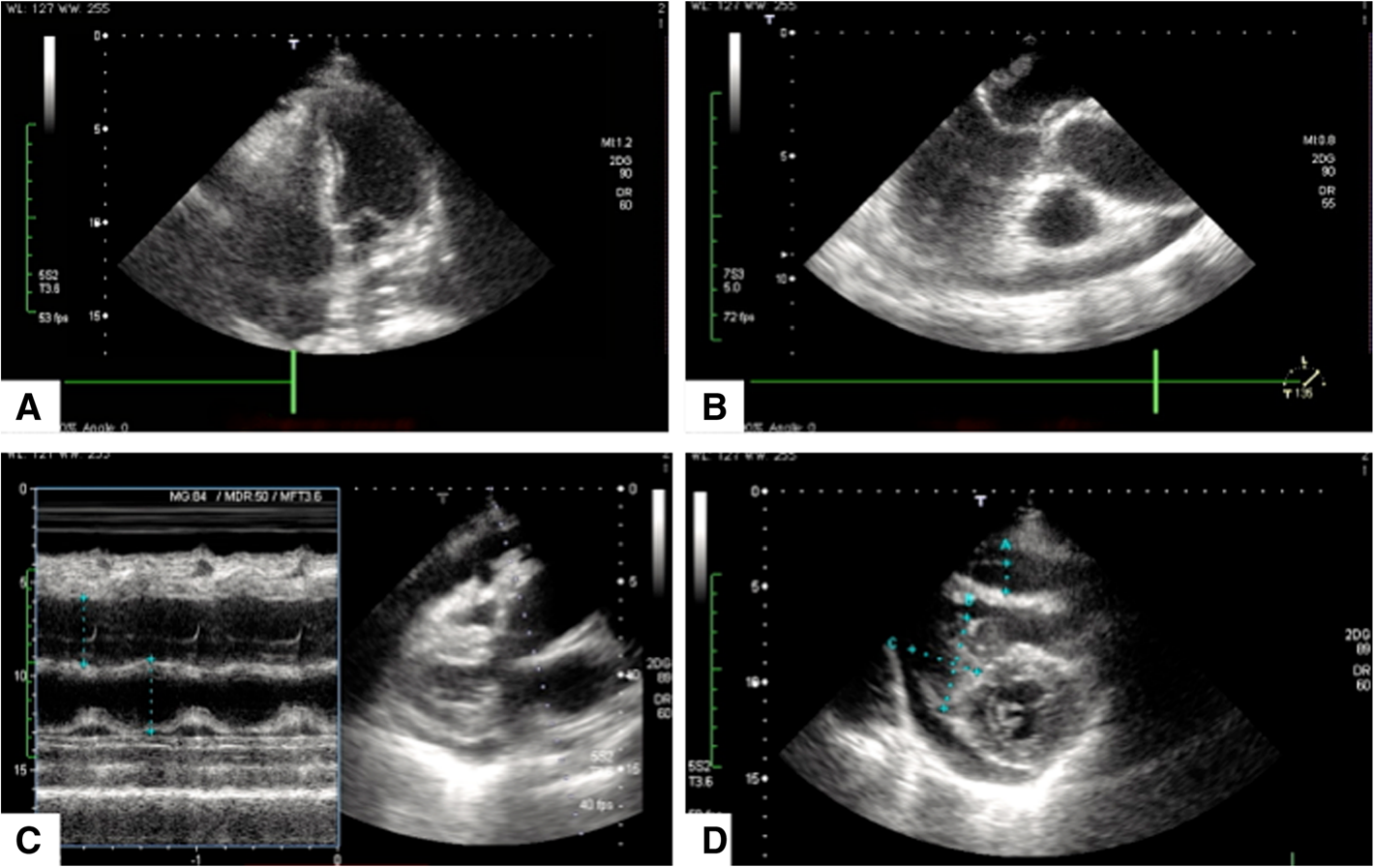
**Initial transthoracic echocardiography (TTE) study showing no structural abnormalities on all views. A** 4-chamber view. Regular ventricles and atria, no suspicious masses can be identified. Intact mitral and tricuspid valvular apparatus. **B** Parasternal long axis showing a regular, non obstructed left ventricular outflow tract. Left Atrium, mitral valve with anterior and posterior leaflet, left ventricle, aortic valve and ascending aorta. No ventricular wall dyskinesias, no obstructions. **C** Aortic root and left atrial diameter measurement. 35,5 mm versus 38.5 mm **D** Parasternal short axis. Mitral valve level showing intact valvular apparatus, no vegetations can be identified.

A 70 y/o immunocompetent Caucasian female presented with palpitations and arrhythmias. The patient’s cardiologist could not detect any structural abnormalities on transthoracic echocardiography (Figure 1A 4-chamber view, Figure 1B parasternal long axis, Figure 1C aortic root and left atrial diameter measurement, Figure 1D parasternal short axis). Within a period of only two weeks she developed progressive dyspnea, facial edema and facial erythema. Transthoracic echocardiography was repeated in our institution and revealed on average 1 cm thick masses on both surfaces of the leaflets of the tricuspid and mitral valves, suggestive of a rapidly progressing underlying pathology. To that point the patient’s past medical history was significant for essential hypertension. There was no history of chest pain, dyspnea or fever. The patient’s cardiac examination revealed no murmurs or extra heart sounds. The patient denied any use of tabaco or alcohol. On physical examination there was a Kussmaul’s sign detectable on inspiration suggestive of an increased jugular venous pressure.

**Figure 2:**
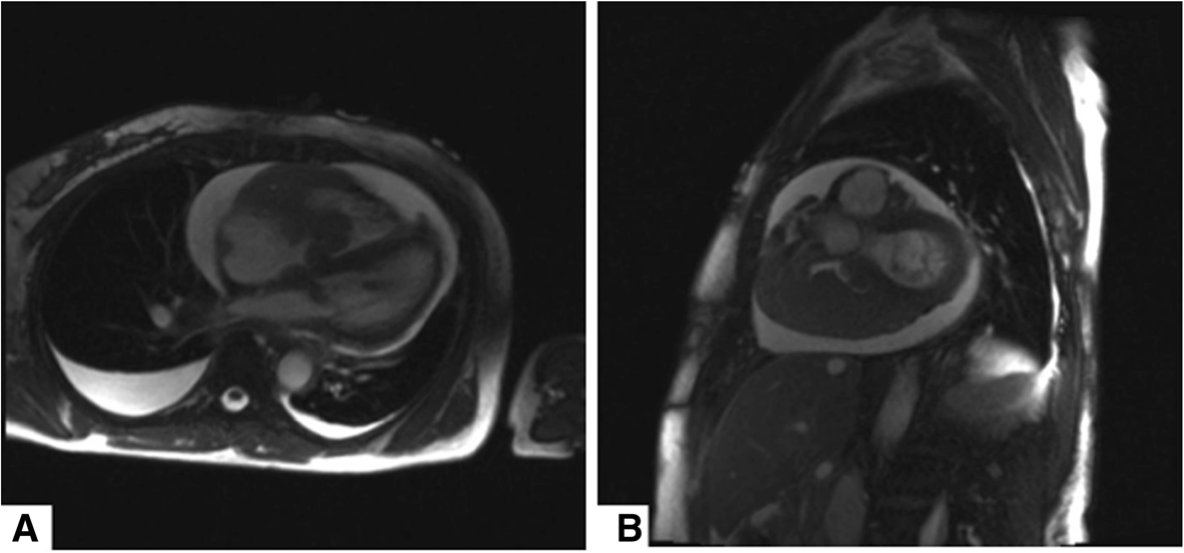
**Magnetic resonance imaging demonstrating no evidence of mediastinal lymphadenopathy. A** Axial view, T2 weighted images showing both pericardial and bilateral pleural effusions, right greater than left. Right ventricular wall irregularities and right greater than left intracavitary tumor formation, partially obstructing the right atrial and the right ventricular flow. **B** Sagittal view, T2 weighted images evidencing intracavitary tumor formation and both pericardial and bilateral pleural effusions. No evidence of mediastinal lymphadenopathy could be observed.

**Figure 3:**
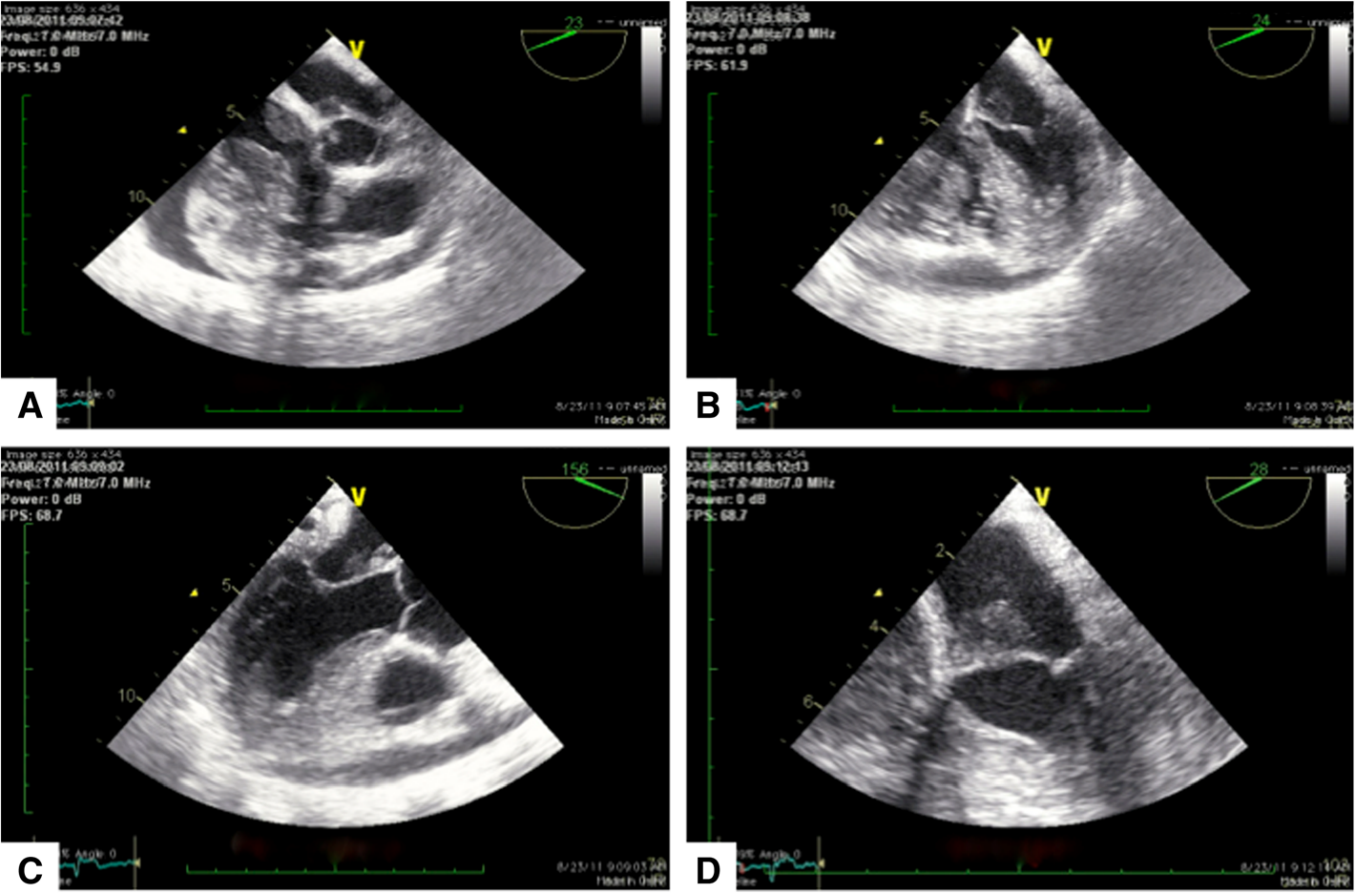
**Repeat transesophageal echocardiography (TEE) study. A** 5-chamber view. Tumor formation in both left and right sided cardiac structures, mitral and tricuspid valves are affected. Of note, both, the right atrium and the right ventricle are significantly obstructed. **B** Mid-esophageal 4-chamber view confirming extensive tumor formation and showing significant involvement of the mitral valve leaflets. Both, the right atrium and the right ventricle are severely obstructed. **C** Mid esophageal long axis confirming mitral valve involvement. **D** 4-chamber view, with cut apex. Tumor formation on mitral valve, however mitral valve apparatus does not appear to be diffusely infiltrated or misconfigured.

**Figure 4:**
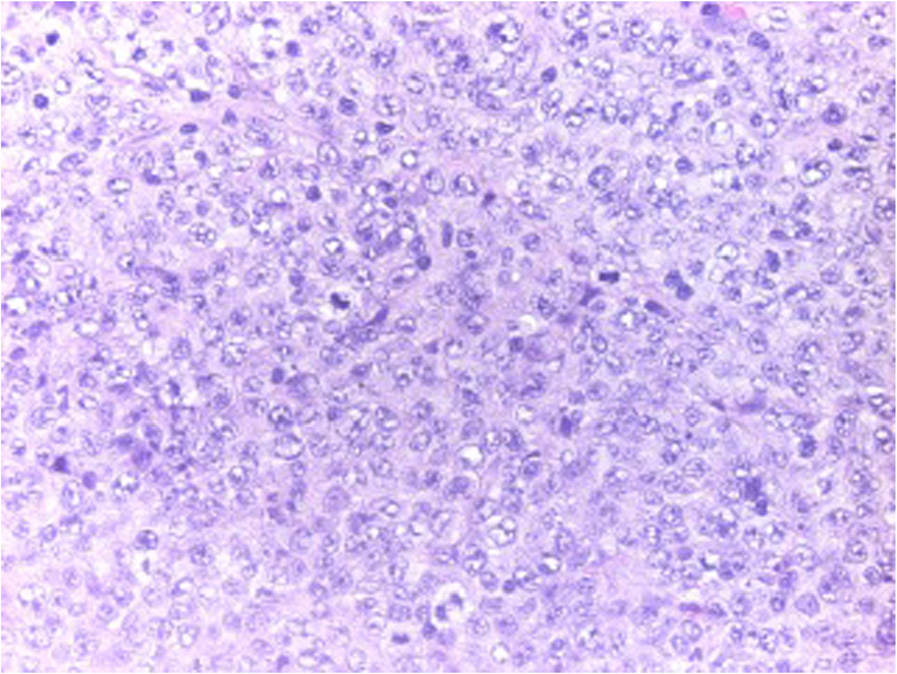
**Histology.** Complete destructive replacement of the myocardial wall by a diffuse large B cell lymphoma that demonstrated plasmacytoid differentiation and blasts.

A magnetic resonance imaging (MRI) scan revealed heterogeneity of the right atrium right ventricle and left atrium and raised concerns of a possible extensive mass (Figure 2A axial view and Figure 2B sagittal view). A transthoracic echocardiogram (TEE) was performed only 3 weeks after the initial transthoracic echocardiography study (Figure 3A 5-chamber view, Figure 3B mid-esophageal 4-chamber view, Figure 3C mid esophageal long axis, Figure 4D, 4-chamber view and additional file 1: Video 1) and revealed a right atrial and right ventricular soft tissue density, diffusely infiltrating both the lateral right atrial wall and free wall of the right ventricle with multisegmental dyskinesias, highly suspicious for a malignant neoplasm. Due to diffuse infiltration the exact tumor dimensions were difficult to calculate, however, the mass was estimated to be 8 cm in total length with an area index of 32 cm^2^ upon four-chamber view. The mass appeared to partially occlude superior vena cava (SVC) and inferior vena cava (IVC) flow but did not extend into either vena cavae. However, both SVC and IVC appeared to be moderately dilated. Due to the extensive growth of the mass right ventricular inflow and outflow tract appeared to be significantly obstructed. There was a functional tricuspid stenosis with a mean gradient of 12 mmHg. Both left atrium and left ventricle were normal in size with an end diastolic left ventricular diameter (LVEDD) of 39 mm, an end systolic left ventricular diameter (LVESD) of 24 mm and a normal systolic function and normal ejection fraction. Of note, there was tumor formation also in the left atrium affecting the mitral valve. Additionally, the patient demonstrated both pericardial effusion of up to 17 mm and bilateral pleural effusions again raising concern of malignant transformation.

A PET scan was performed demonstrating pathologic fluorodeoxyglucose (^18^ F-FDG) tracer uptake in the right atrial wall, right ventricular wall and left atrium. Coronary angiography revealed no abnormalities.

Due to pending SVC obstruction, the patient underwent rapid surgical excision of the mass in open-heart surgery. The operation was performed with inferior vena cava and anonymous vein cannulation due to the fact that the SVC was obstructed and a left ventricular vent via the right superior pulmonary vein on cardiopulmonary bypass. Myocardial protection was achieved with antegrade aortic cardioplegia only. Both the right atrial and right ventricular walls and cavitites consisted of dense, fibrotic composition and tumor masses were palpable along the right side of the heart. Intraoperatively no lumen was detected in the right atrium. Only surgical debulking was feasible as the entire right side of the heart appeared to consist only of tumor mass. In order to create a new right atrial cavity a pericardial patch was implanted and sewed to the right atrial margins by means of 5–0 prolene suture.

The excised masses were of soft and homogeneous composition. Frozen sections were sent for histopathologic evaluation and revealed complete destructive replacement of the myocardial wall by a diffuse large B cell lymphoma that demonstrated plasmacytoid differentiation (non-Hodgkin’s lymphoma, Figure 4).

Further histological evaluation of morphology, immunohistochemistry, conventional cytogenetics and in-situ hybridization revealed high-grade CD20+, CD79a+, BCL-2+ BCL-6+ and MUM-1+ blasts. Only 10% of tumor cells showed c-MYC immunoreactivity. An additionally performed in-situ hybridization for Epstein Barr Virus (EBV) was negative.

Following surgery there was a grade I tricuspid insufficiency. The patient was off all inotropes and pressors on postoperative day 2 and eventually discharged for further oncologic treatment in good clinical condition with normal ejection fraction and good right ventricular function. The patient was started on a standard chemotherapy protocol of 6 cycles rituximab, cyclophosphamide, doxorubicin, vincristine and prednisone (R-CHOP). However, despite the highly malignant nature of the lymphoma the regimen was initiated only after a time span of 2 weeks after surgery due to major concerns of potential ventricular rupture as the neoplasm was diffusely infiltrating cardiac structures including the entire free wall of the right ventricle. Post-surgical adhesions between the heart and the pericardial sac were thought to prevent major bleedings beyond this 2-week period.

## Discussion

Primary cardiac lymphomas are rare neoplasms and comprise 1.3% of all cardiac tumors [3]. To date, fewer than 90 tumors have been described in literature, mostly as single case reports [5].

Primary cardiac lymphomas must be distinguished from other primary malignant tumors of the heart such as angiosarcomas (the most common primary malignancy of the heart) and from the more common benign cardiac tumors such as myxomas and lipomas. Because diffuse large B cell lymphomas, are highly malignant and aggressive neoplasms, prompt diagnosis and therapy (chemotherapy and surgery) are key factors for favorable clinical outcomes. Of note, diffuse large B cell lymphomas are extremely fast growing neoplasms. As in the case of our patient no structural abnormalities could be detected upon the initial transthoracic echocardiography study, however, manifestation of the tumor progressed to both impressive and dramatic dimensions within only two weeks’ time and prompted for rapid surgery.

Cardiac tumors have been called the great mimickers, symptom-wise and diagnostic-wise. Symptom-wise, no pathognomic features exist and cardiac neoplasms may present with chest pain, arrhythmias, superior vena cava syndrome if in an intracavitary localization and obstructing venous flow, thrombus formation and peripheral embolism, intractable heart failure, pericardial effusion or sudden cardiac death and classic constitutional symptoms such as fever, malaise and weight loss. Diagnostic-wise, presentation of cardiac lymphoma on imaging studies may not be unambiguous since they potentially mimic other cardiac neoplasms including myxomas, angiosarcoma or rhadomyomas. Therefore, diagnosis of cardiac tumors vitally depends on the use of multiple imaging techniques, including cardiac magnetic resonance imaging (MRI), cardiac computed tomography (CT), positron emission tomography (PET) or fusion of the latter two (PET-CT). This imaging approach is complemented with the use of endomyocardial biopsy, excisional intraoperative biopsy and pericardial fluid cytological evaluation to establish definitive diagnosis.

## Conclusion

When possible, surgical excision in combination with systemic chemotherapy remains the gold standard of treatment for primary malignant cardiac tumors. Cardiac tumors are the great mimickers; therefore multimodality cardiac imaging is the key to early diagnosis and favorable patient outcome.

## Consent

Written informed consent was obtained from the patient for publication of this case report and any accompanying images. A copy of the written consent is available for review by the Editor-in-Chief of this journal.

## Abbreviations

BCL-2/6: B cell lymphoma; c-MYC: Myelocytomatosis oncogene; CD20/79a: Cluster of differentiation; CT: Computed tomography; EBV: Epstein Barr Virus; EF: Ejection fraction; F-FDG: Fluorodeoxyglucose; MUM-1: Multiple myeloma oncogene; MRI: Magnetic resonance imaging; PET: Positron emission tomography; R-CHOP: Rituximab, cyclophosphamide, doxorubicin, vincristine and prednisone; TOE: Transesophageal echocardiography; y/o: Years old.

## Competing interests

The authors declare that they have no competing interests.

## Authors’ contributions

AH, assisted during surgery and prepared the manuscript. ME, aided in literature search, participated in surgery. DW, aided in literature search. BM, performed the echocardiography studies. CR, aided in literature search. AK, operating surgeon, drafted and reviewed the manuscript. All authors read and approved the final manuscript.

## Authors’ information

AH PhD student, Department of Cardiac Surgery, Vienna General Hospital, Medical University of Vienna.

ME professor of surgery, Department of Cardiac Surgery, Vienna General Hospital, Medical University of Vienna.

BM attending physician, Department of Cardiothoracic and Vascular Anesthesia and Intensive Care Medicine, Vienna General Hospital, Medical University of Vienna.

CR PhD student, Department of Cardiac Surgery, Vienna General Hospital, Medical University of Vienna.

DW chief-resident, Department of Cardiac Surgery, Vienna General Hospital, Medical University of Vienna.

AK professor of surgery, Department of Cardiac Surgery, Vienna General Hospital, Medical University of Vienna.

## Supplementary Material

Additional file 1: Video 1 5-chamber view evidencing tumor formation in both left and right sided cardiac structures. Of note, both, the right atrium and the right ventricle are obstructed. Click here for file
